# Supplementary material for: Effect of liver biopsy size on MASLD fibrosis assessment by second-harmonic generation/two-photon excitation fluorescence microscopy
Source: JHEP Rep. 2025 May 8;7(8):101449. doi: 10.1016/j.jhepr.2025.101449 (PMC12260414; doi:10.1016/j.jhepr.2025.101449)
Supplement: Multimedia component 1 [file mmc1.pdf]

Effect of liver biopsy size on MASLD fibrosis assessment by second harmonic generation/two-photon excitation fluorescence microscopy

Daniel T. Field, Yayun Ren, Kutbuddin Akbary, Elaine Chng, Dean Tai, Nikolai V. Naoumov, David E. Kleiner, Jonathan A. Fallowfield, Timothy J. Kendall, Arun J. Sanyal

Table of contents

Fig. S1……………………………………………………………………………………2

Fig. S2……………………………………………………………………………………3

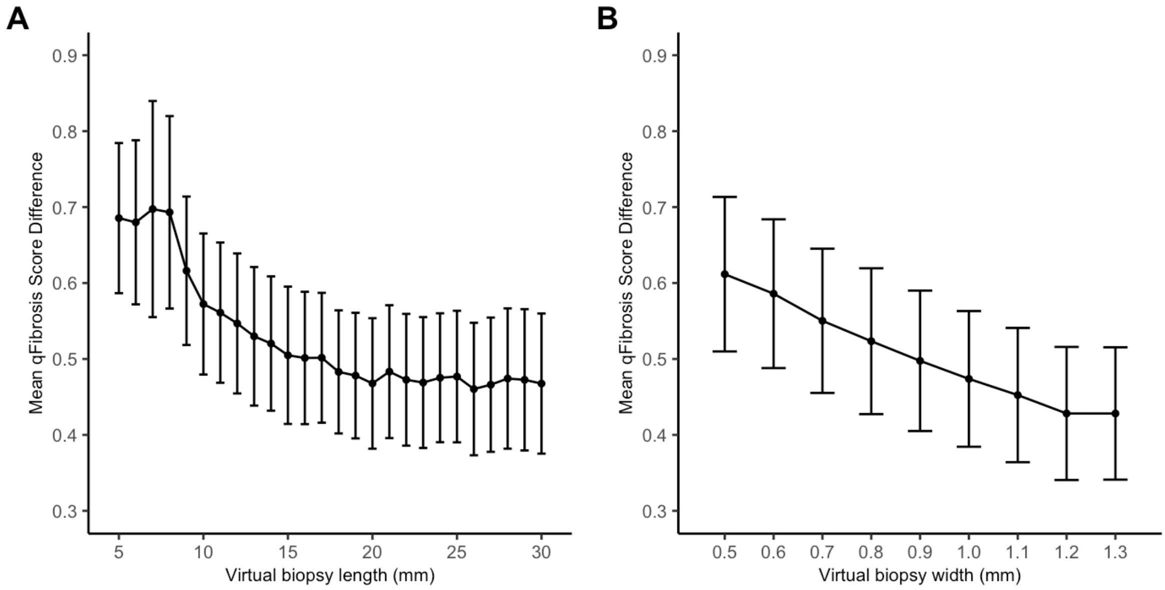

Fig. S1. qFibrosis continuous value mean absolute differences between virtual biopsies and whole parent section*. (A) Across length variation. (B) Across width variation. Mean with standard error of the mean.*

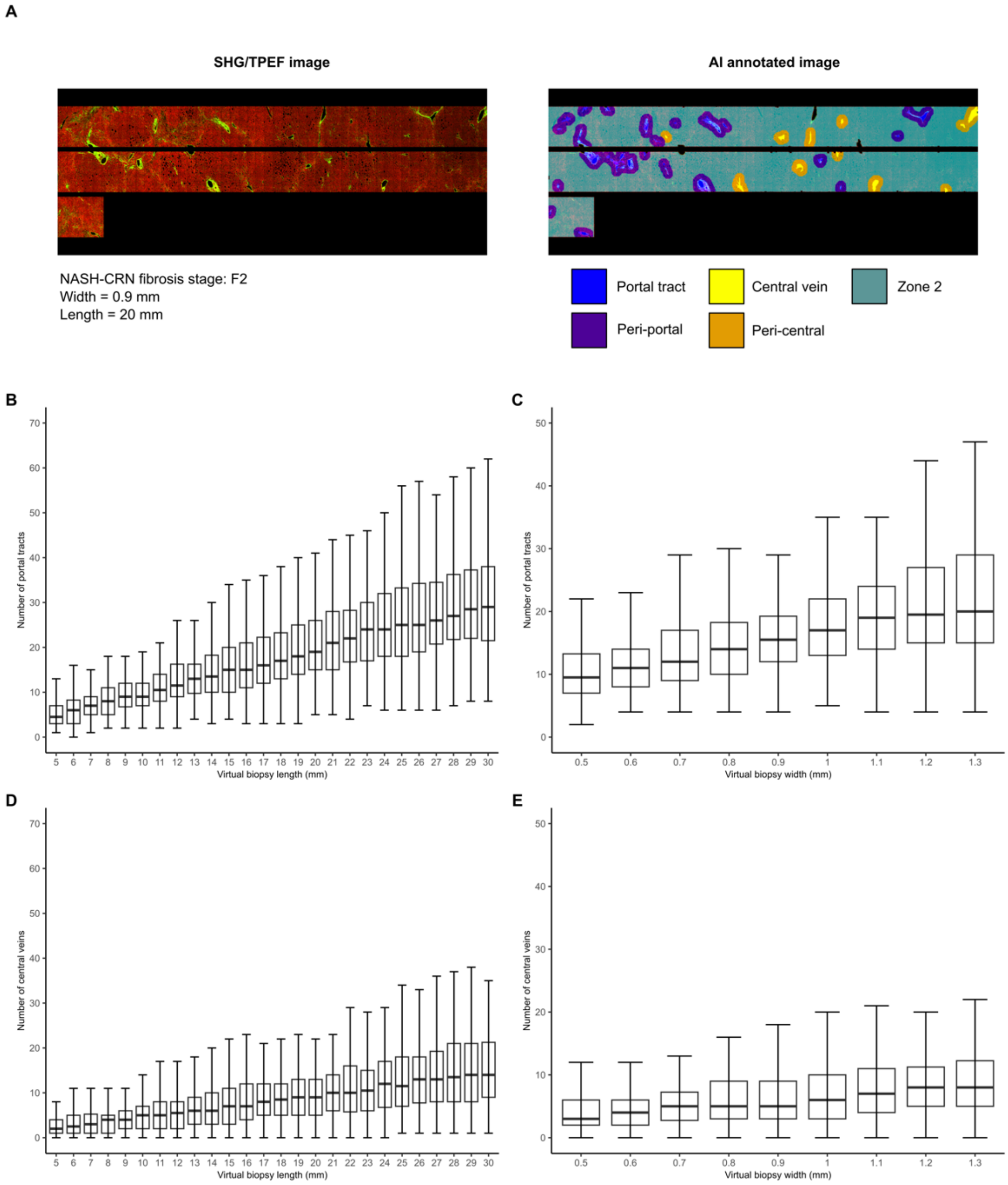

Fig. S2. Portal tract and central vein number in virtual biopsies from cases across all pathologist-assigned NASH-CRN stages. *(A) AI segmentation of central vein and portal vein areas. (B) Portal tracts across length variation. (C) Portal tracts across width variation. (D) Central veins across length variation. (E) central veins across width variation. All boxplots are median with lower and upper hinges corresponding to the first and third quartiles, whiskers extend to values no further than 1.5 * inter-quartile range from the hinge.*
