# Supplementary material for: Effect of liver biopsy size on MASLD fibrosis assessment by second-harmonic generation/two-photon excitation fluorescence microscopy
Source: JHEP Rep. 2025 May 8;7(8):101449. doi: 10.1016/j.jhepr.2025.101449 (PMC12260414; doi:10.1016/j.jhepr.2025.101449)
Supplement: Multimedia component 4 [file mmc4.pdf]

Effect of liver biopsy size on MASLD fibrosis assessment by second-harmonic generation/two-photon excitation fluorescence microscopy

Daniel T. Field[1,†], **Yayun Ren**[2,†], Kutbuddin Akbary[2], Elaine Chng[2], Dean Tai[2], Nikolai V. Naoumov[3], David E. Kleiner[4], Jonathan A. Fallowfield[5], Timothy J. Kendall[1,5,*], Arun J. Sanyal[6]

JHEP Reports **2025**. vol. 7 | 1–11

Check for updates

Background & Aims: Fibrosis in metabolic dysfunction-associated steatotic liver disease (MASLD) is a prognostic indicator and clinical trial efficacy endpoint. Second-harmonic generation/two-photon excitation fluorescence (SHG/TPEF) microscopy images unstained tissue sections and, when integrated with artificial intelligence models, generates a continuous fibrosis value (qFibrosis) and ordinal qFibrosis stage. The impact of biopsy size and location on the accuracy of these approaches has not been assessed in MASLD, leaving quality assurance procedures undefined.

Methods: One unstained section each from 100 hepatectomy/explant MASLD cases, 20 of each pathologist-assigned Non-alcoholic Steatohepatitis Clinical Research Network (NASH-CRN) fibrosis stage (F0–F4), were used to create virtual core biopsies by cropping regions from within the whole parent section. Regions varied in length (5–30 mm) with a fixed width of 0.9 mm, width (0.5–1.3 mm) with a fixed length of 15 mm, or position within the whole parent section. SHG/TPEF was used, and the qFibrosis continuous value and stage of the virtual core biopsies were determined for comparison with those of the whole parent section.

Results: The qFibrosis continuous value and stage correlated strongly with pathologist-assigned NASH-CRN stage (r_s = 0.92). Increasing the length and width of virtual biopsies increased the correlation between the qFibrosis continuous value and the agreement with the qFibrosis stage of the whole parent section, stabilising between 20–26 mm in length and 0.9 mm in width. The position within the tissue did not influence qFibrosis metrics.

Conclusions: Longer (>20 mm) and wider (>0.9 mm) biopsies provide more accurate fibrosis assessment using SHG/TPEF. Biopsy position and orientation do not influence accuracy.

© 2025 The Author(s). Published by Elsevier B.V. on behalf of European Association for the Study of the Liver (EASL). This is an open access article under the CC BY license (http://creativecommons.org/licenses/by/4.0/).

Introduction

Metabolic dysfunction-associated steatotic liver disease (MASLD) is the leading cause of chronic liver disease worldwide and now affects an estimated 38% of the population.[1] The histological spectrum includes fat in the absence of inflammation (simple steatosis) and metabolic dysfunction-associated steatohepatitis (MASH), characterised by inflammation and hepatocellular ballooning, that drives the development of fibrosis, cirrhosis, and primary liver cancer.[2]

Regulatory bodies require ordinal scoring of disease activity and fibrosis and consider improvement in those scores as a meaningful surrogate endpoint.[3,4] However, observer-based subjective scoring is inherently compromised by inter- and intra-observer variation.[5] The widespread adoption of digital pathology has facilitated the development of observer-independent tools for feature quantification and scoring,[6,7] although the use of stained sections still requires that staining variation be accounted for. Second-harmonic generation/two-photon excitation fluorescence (SHG/TPEF) microscopy enables the quantification of fibrospatial features on unstained sections.[8] The qFibrosis continuous value derives from multiple imaged collagen parameters and can be converted to a semi-quantitative qFibrosis score.[9] The qFibrosis score has been used to assess fibrosis changes in clinical trials (*e.g.* MAESTRO-NASH phase III [NCT03900429] and FLIGHT-FXR phase IIa/b [NCT02855164])[10,11] and can also predict clinical outcomes.[12,13] Moreover, a recent study has demonstrated that pathologists using the SHG/TPEF-based artificial intelligence (AI)-assisted analytical tools show increased agreement in MASH fibrosis staging.[14]

The heterogeneity of histological features indicates that short, fragmented samples often introduce sampling variability.[15] In two MASLD studies, longer total biopsy length was associated with increased fibrosis score, implying that shorter

* Corresponding author. Address: Centre for Inflammation Research, Institute for Regeneration and Repair, University of Edinburgh, 5 Little France Drive, Edinburgh, EH16 4UU, UK. Tel.: +44-0131-2427007.
E-mail address: Tim.Kendall@ed.ac.uk (T.J. Kendall).
† These authors contributed equally.
https://doi.org/10.1016/j.jhepr.2025.101449

samples systematically underestimate fibrosis.[15,16] Core width also affects fibrosis assessment, with narrower cores leading to underscoring of fibrosis.[17] Clinical guidelines, therefore, recommend a 20-mm-length core using a 16G needle, although many routine care and clinical trial biopsies fall short of these recommendations.[18]

Although the criteria for liver biopsy quality required for traditional pathological assessment are well established, there are no data defining biopsy adequacy for computational assessment. Furthermore, an additional section complementing a Masson's trichrome (or other matrix stain) is required in studies using SHG/TPEF, but there are limited data documenting the potential difference in scar amount in adjacent sections that such a requirement introduces. To address this, virtual cores from large resection or explant sections were created to assess the impact of biopsy length, width, core adjacency (analogous to the additional 'adjacent' unstained section used for SHG/TPEF imaging), and rotation on fibrosis evaluation using SHG/TPEF and define a standard for liver biopsies in clinical trials where such imaging is used.

Patients and methods

Patient cohort and tissue selection

A single unstained 4-μm section from each of 100 explant or resection cases from the previously described SteatoSITE MASLD cohort[19] was used (Supplementary CTAT Table). Briefly, inclusion criteria were as follows: men or women; >18 years of age at the time of tissue sampling; all ethnic groups, socioeconomic backgrounds, and health status; and dead or alive at the time of inclusion into the data commons. Cases were excluded if any of the following applied: history of chronic liver disease of any non-MASLD aetiology or excessive alcohol (>21 units per week for men, >14 units per week for women) consumption on specimen request or histological features indicating a secondary non-MASLD diagnosis. Non-alcoholic steatohepatitis Clinical Research Network (NASH-CRN) fibrosis stages for each were available as part of the SteatoSITE dataset, and n = 20 of each pathologist-assigned NASH-CRN fibrosis stage (F0–F4) were randomly selected.

The ethical approvals to create the SteatoSITE resource have been previously described.[19] Anonymized tissue was supplied after approval by the National Health Service Research Scotland (NRS) Biorepository Network (Reference: SR1032; 2 August 2018). Unified transparent approval for unconsented data inclusion was provided by the West of Scotland Research Ethics Committee 4 (Reference: 20/WS/0002; 18 February 2020), the Public Benefit and Privacy Panel for Health and Social Care (PBPP; Reference: 1819-0091; 4 June 2021), institutional research and development departments, and Caldicott Guardians. Approval for the current study was given by the SteatoSITE Scientific Advisory Board after application and review (ST001, 13 September 2021).

SHG/TPEF microscopy and qFibrosis metric generation

Unstained formalin-fixed paraffin-embedded sections were deparaffinized, and stain-free tissue scanning was performed on a Genesis®200 imaging system (HistoIndex Pte. Ltd., Singapore) as previously described.[20,21] To provide a stain-free assessment of fibrosis, specimens were femto-laser-excited at 780 nm with resultant SHG signals detected at 390 nm. SHG photons (of half the wavelength of the original source) are exclusively produced from interactions with non-centrosymmetric structures within tissue, notably fibrillary collagen. The intensity and spatial distribution of the SHG signals can therefore accurately map the extent and location of hepatic fibrosis. The sensor-detected TPEF signals at 550 nm (within the visible range) following dual photon tissue excitation and subsequent emittance allow mapping of background structures. Images were acquired at 20× magnification with a 512 × 512-pixel resolution.

Multiple adjacent image tiles (at 200 × 200 μm) were taken to incorporate the entire tissue on the slide, regarded herein as the 'whole parent section'. The resulting image distinguishes the fibrosis in green and the surrounding structures in red (Fig. 1). The qFibrosis continuous value was calculated using AI-based algorithms of captured images that use 184 previously validated parameters of hepatic fibrosis.[9,13] This, in turn, can be segmented into equivalent fibrosis stages F0–F4 (qFibrosis stage) based on pre-established cut-offs.

Virtual biopsy creation

The pathologist-assigned NASH-CRN fibrosis stage supplied with the SteatoSITE images is derived from the whole parent section, and the qFibrosis continuous value is derived from the SHG/TPEF images of the whole parent section. These values were regarded as the 'ground truth'. To determine how the qFibrosis metrics would be affected by biopsy sampling variation, virtual core biopsies were created from each of the SHG/TPEF images through an automated 'cropping' process involving variable lengths, widths, and positions, using the approach developed by Bedossa *et al.*[22]

For length variation, a fixed width of either 0.5 or 0.9 cm was used, and length increased in increments of 1 mm from 5 mm up to 30 mm. If a desired length exceeded the tissue dimension and cannot be captured in one pass (*i.e.* the virtual biopsy is 25 mm, but the tissue is only 20 mm in maximum dimension), the virtual biopsy was continued approximately 0.25 mm below the first 'pass' to produce a biopsy of the desired length. The automated system used a threshold of non-zero pixels to determine when the next line started. For width variation, a fixed length of 15 mm was chosen, and width increased in increments of 1 mm from 0.5 mm up to 1.3 mm. Values and increments were chosen roughly from the expected spread of lengths and widths seen in standard 18G and 16G needle liver biopsies.[23] Using an AI-based algorithm, portal tracts and central veins were identified and summed, based on the SHG/TPEF images of each virtual core biopsy, as previously described.[21]

Because SHG/TPEF requires an unstained section in addition to the routine matrix- and H&E-stained sections, adjacent position analysis was conducted to model this requirement and determine whether an alternative position in the block would affect the qFibrosis metrics. A fixed length of 15 mm and a width of 0.9 mm were used, with the analysis performed on paired, immediately adjacent, cropped tissue (Fig. 1).

Finally, to assess the robustness of the qFibrosis metric to positional variations that may be encountered during biopsy obtainment, the virtual biopsies were rotated from 0° up to 160° in an anticlockwise direction at increments of 20°, maintaining a constant length of 15 mm and width of 0.9 mm.

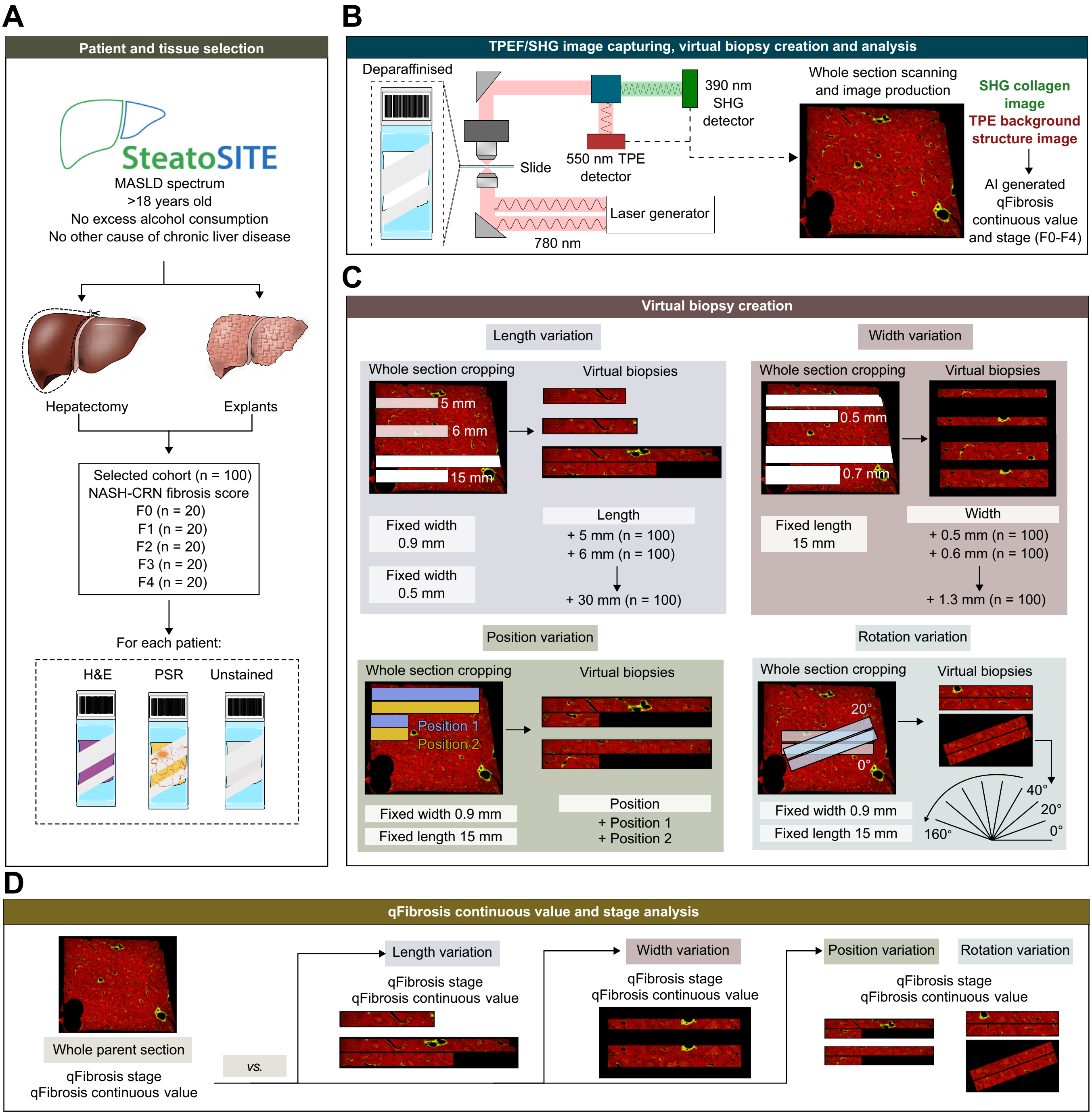

Fig. 1. Schematic of study methodology. (A) Twenty resection or explant cases of each pathologist-assigned NASH-CRN fibrosis stage from the SteatoSITE cohort of patients with MASLD were included. An unstained slide from each of these patients who underwent either explant or hepatectomy was selected for SHG/TPEF microscopy. (B) The unstained slide is de-paraffinized and laser-excited at 780 nm. The resultant SHG signal is detected at 390 nm, and the TPEF signal is detected at 550 nm. The slide is scanned in 200 × 200 µm segments to produce a whole-slide image of the tissue (whole parent section). TPE signals, capturing collagen, are in green, and the remaining tissue is in red. AI-assisted qFibrosis assessment of the whole image is calculated as a continuous value and adjusted ordinal qFibrosis stage. (C) The whole parent section is then cropped for variations in length (fixed width 0.9 mm and variable length between 5 and 30 mm in increments of 1 mm), width (fixed length of 15 mm and variable width between 0.5 and 1.3 mm in increments of 0.1 mm), and adjacent positioning. (D) The qFibrosis continuous value and qFibrosis stage are computed for each sampling variation and compared against the whole parent section. AI, artificial intelligence; MASLD, metabolic dysfunction-associated steatotic liver disease; NASH-CRN, Non-alcoholic Steatohepatitis Clinical Research Network; PSR, Picrosirius Red; SHG, second-harmonic generation; TPEF, two-photon excitation fluorescence.

Statistical analysis

The correlation between qFibrosis continuous values and pathologist-assigned NASH-CRN stage was determined using Spearman's correlation. Spearman's correlation coefficient (r_s) was also calculated for each variation in biopsy length, width, and position by comparing the qFibrosis continuous value of each virtual core biopsy against the qFibrosis continuous value derived from the whole parent section.

To assess consistency and variation in the qFibrosis continuous value across biopsy sizes, the coefficient of variation (CoV) of the qFibrosis continuous value was calculated for each length and width of the virtual core biopsies. The CoV is calculated as the ratio of the standard deviation to the mean of the qFibrosis continuous value for a given biopsy length or width (presented as a percentage).[22] The lower the CoV, the less the variation there is in the qFibrosis continuous value at that length or width.

Agreement between the ordinal qFibrosis stage derived from the whole parent section or virtual cores and pathologist-assigned NASH-CRN stage was compared using linear weighted kappa. Statistical significance was defined at the 95% confidence level (p <0.05). The analyses were performed using MATLAB software (version R2021b; The MathWorks, Inc., Natick, MA, USA).

Results

Baseline correlation of qFibrosis metrics from the whole parent section with pathologist-assigned NASH-CRN fibrosis stage

There was a strong correlation between the qFibrosis continuous value derived from SHG/TPEF imaging of the whole parent section and the pathologist-assigned NASH-CRN fibrosis stage (r_s = 0.92, p <0.001; Fig. 2A). Similarly, the qFibrosis stage derived from imaging the whole parent section showed good concordance with the pathologist-assigned NASH-CRN stage (linear weighted kappa 0.78, SE 0.037, p <0.01; Fig. 2B).

Increasing virtual biopsy length increases agreement between virtual biopsy-derived qFibrosis metrics and whole parent section-derived metrics and fibrosis stage

For all virtual biopsy lengths tested at a fixed width of 0.9 mm (Fig. 3A), the Spearman's rank correlation coefficient between virtual core biopsy-derived qFibrosis continuous value and whole parent section-derived qFibrosis continuous value remained above 0.8 (Fig. 3B). Increasing the virtual biopsy lengths enhanced the correlation between virtual core biopsy-derived and whole parent section-derived qFibrosis continuous values up to a plateau at approximately 23 mm (r_s = 0.94). Any increment in biopsy length beyond this point had minimal effect on correlation.

Similarly, as biopsy length increased, the CoV of the qFibrosis continuous values at each NASH-CRN fibrosis stage decreased (Fig. 3C). For all fibrosis stages, the CoV was 18% at a length of 5 mm, 13% at 15 mm, and 11% at 30 mm, with a notable plateau at ~26 mm. Beyond this length, there was limited effect on variability. Greater variation was seen in NASH-CRN pathologist-assigned F4 cases (CoV of 27% at 5 mm and 20% at 30 mm), but in stages F0–F3, the variation was lower and comparable between stages (Fig. 3C). The absolute mean difference between the qFibrosis continuous value derived from the virtual core biopsies or whole parent section at each length also decreased as length increased, demonstrating improved consistency in metrics as length extended (Fig. S1A).

When comparing the qFibrosis stage derived from virtual core biopsies with the pathologist-assigned NASH-CRN stage of the whole parent section, the linear weighted kappa also increased with biopsy length (kappa >0.6 for all lengths, 0.61 at 5 mm, 0.65 at 15 mm, and 0.72 at 30 mm), with a plateau above 20 mm in length (Fig. 3D).

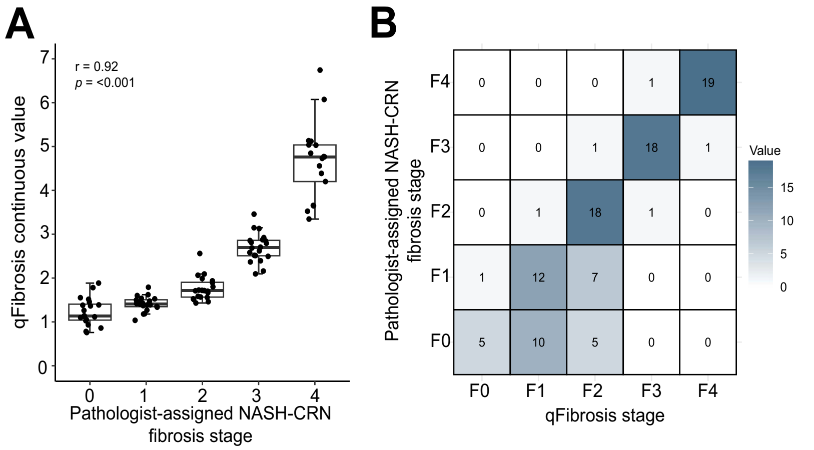

Fig. 2. Performance of qFibrosis continuous value and stage on whole parent section. (A) Correlation of using whole parent section of qFibrosis continuous value with pathologist-assigned NASH-CRN fibrosis stage. Spearman's correlation coefficient = 0.92, p <0.001. Tukey's fences method was used to exclude outliers from the plot (n = 3). Boxplots are median with lower and upper hinges corresponding to the first and third quartiles, respectively, and whiskers extend to values no further than 1.5× IQR from the hinges. (B) Confusion matrix of qFibrosis stage against pathologist-assigned NASH-CRN fibrosis stage. NASH-CRN, Non-alcoholic Steatohepatitis Clinical Research Network.

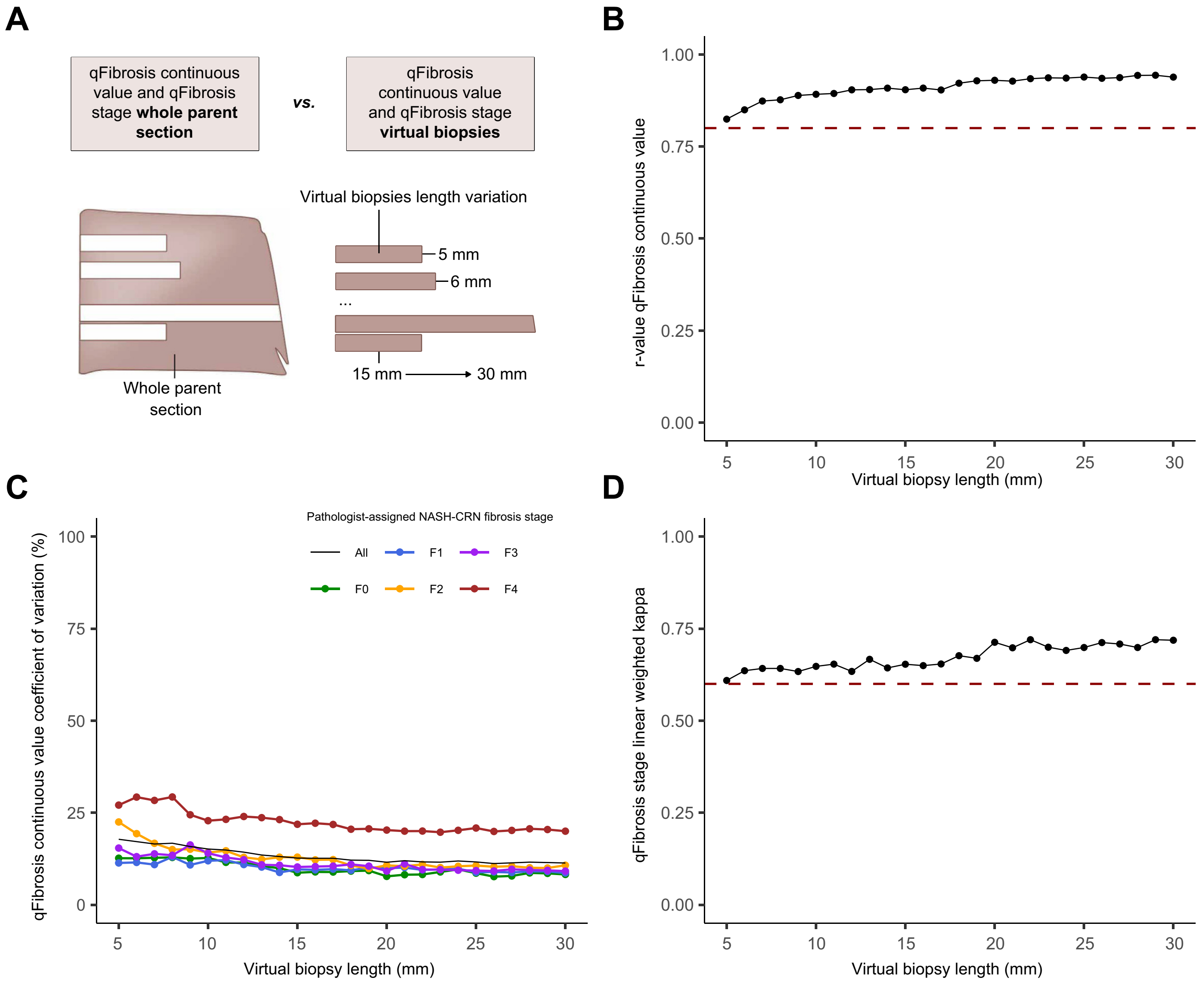

Fig. 3. Influence of length sampling variation on qFibrosis metrics. (A) Schematic of methodology for assessing virtual core biopsy length variation. (B) Spearman's correlation coefficients between the qFibrosis continuous value of virtual core biopsies of varying length and the qFibrosis continuous value of the whole parent section. Red dotted line at Spearman's correlation coefficient r_s = 0.8. (C) Coefficient of variation for the qFibrosis continuous value for virtual core biopsy of varying lengths and the pathologist-assigned NASH-CRN fibrosis stage of the whole parent section. (D) Linear weighted kappa scores comparing qFibrosis stage of virtual core biopsies of varying lengths and the NASH-CRN stage of the whole parent section. Red dotted line at a linear weighted kappa of 0.6. NASH-CRN, Non-alcoholic Steatohepatitis Clinical Research Network.

The number of portal tracts and central veins increased as virtual biopsy length increased, as expected (Fig. S2B and D). At a length of 20 mm, a mean of 21 portal tracts and 10 central vein profiles were identified in each core.

Increasing virtual core biopsy width increases agreement between virtual core biopsy-derived qFibrosis metrics and whole parent section-derived metrics and fibrosis stage

Similar to the observed effect of length variation, the correlation between virtual core biopsy-derived qFibrosis continuous value and whole parent section-derived qFibrosis continuous value increased as virtual biopsy width increased (Fig. 4A), with Spearman's correlation coefficient values 0.9 or above, indicating a robust agreement (Fig. 4B). At 0.5 mm width, r_s was 0.90 and increased to 0.94 at 1.3 mm, although the value stabilised at 1 mm.

For all NASH-CRN pathologist-assigned stages of fibrosis, the CoV for the qFibrosis continuous value progressively decreased as the width of virtual core biopsies increased, from 17% at 0.5 mm, 12% at 0.9 mm, and 10% at 1.3 mm (Fig. 4C). When accounting for the stage of fibrosis, similar to length, cases with NASH-CRN pathologist-assigned F4 fibrosis showed a higher CoV (27% at 0.5 mm and 17% at 1.3 mm) compared with F0–F3 cases. Nevertheless, a consistent decline in CoV was evident as the width increased. Cases with NASH-CRN pathologist-assigned F0–F3 fibrosis showed lower CoV but also demonstrated a trend towards higher CoV as fibrosis advanced. The absolute mean difference in qFibrosis continuous value derived from virtual core biopsy or whole parent section decreased as the width increased (Fig. S1B).

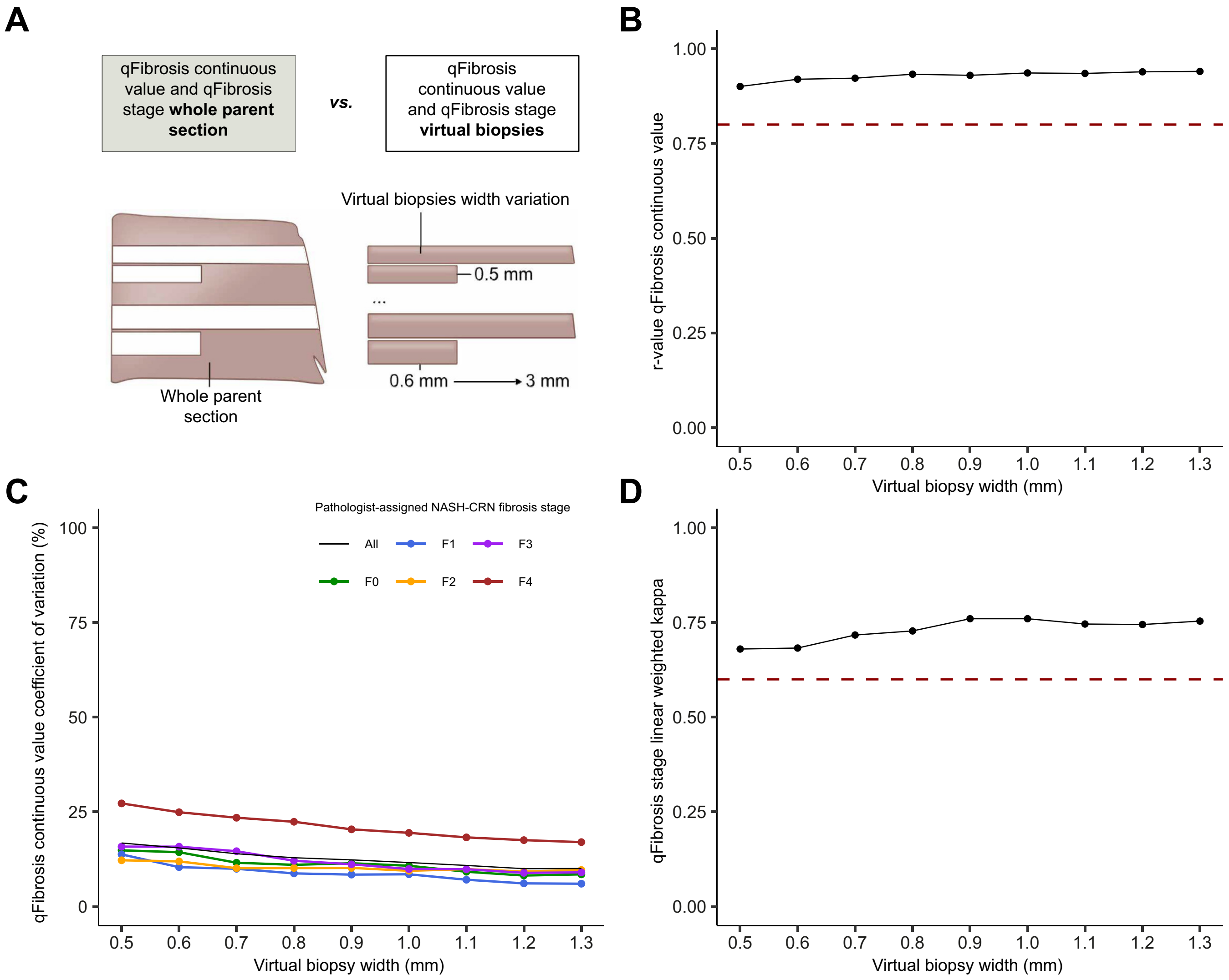

Fig. 4. Influence of width sampling variation on qFibrosis metrics. (A) Schematic of methodology for assessing virtual core biopsy width variation. (B) Spearman's correlation coefficients between the qFibrosis continuous value of virtual core biopsies of varying widths and the qFibrosis continuous value of the whole parent section. Red dotted line at Spearman's correlation coefficient r_s = 0.8. (C) Coefficient of variation for the qFibrosis continuous value for virtual core biopsy of varying widths and pathologist-assigned NASH-CRN fibrosis stage of the whole parent section. (D) Linear weighted kappa scores comparing qFibrosis stage of virtual core biopsies of varying widths and the NASH-CRN stage of the whole parent section. Red dotted line at a linear weighted kappa = 0.6. NASH-CRN, Non-alcoholic Steatohepatitis Clinical Research Network.

The linear weighted kappa for the agreement between the qFibrosis stage derived from the virtual core biopsy or whole parent section also improved with increasing biopsy width. Kappa values were above 0.6 (Fig. 4D); at a virtual core biopsy width of 0.5 mm, the linear weighted kappa was 0.68, increasing to 0.76 at 0.9 mm (the apparent plateau).

The number of portal tracts and central veins increased as the virtual biopsy width increased, as expected (Fig. S2C and E). At a width of 0.9 mm, a mean of 17 portal tracts and six central vein profiles were identified in each core.

qFibrosis continuous value and stage are robust to virtual core biopsy position

The qFibrosis continuous values derived from virtual core biopsies from adjacent positions in the whole parent section (Fig. 5A) showed consistent correlations with the NASH-CRN pathologist-assigned stages (Fig. 5B). The correlations between qFibrosis continuous values derived from each adjacent position and the correlation between the qFibrosis continuous value derived from the adjacent positions and the whole parent section were similar and high, with r_s values of 0.92, 0.94, and 0.92 for correlation between positions 1 and 2, position 2 and the whole parent section, and position 1 and the whole parent section, respectively (Fig. 5C).

The agreement between qFibrosis stage derived from virtual core biopsies in adjacent positions, qFibrosis stage derived from the whole parent section, and the NASH-CRN pathologist-assigned stage was also similar (Fig. 5D). The linear weighted kappa value for agreement between qFibrosis stages derived from adjacent virtual core biopsy positions was 0.71.

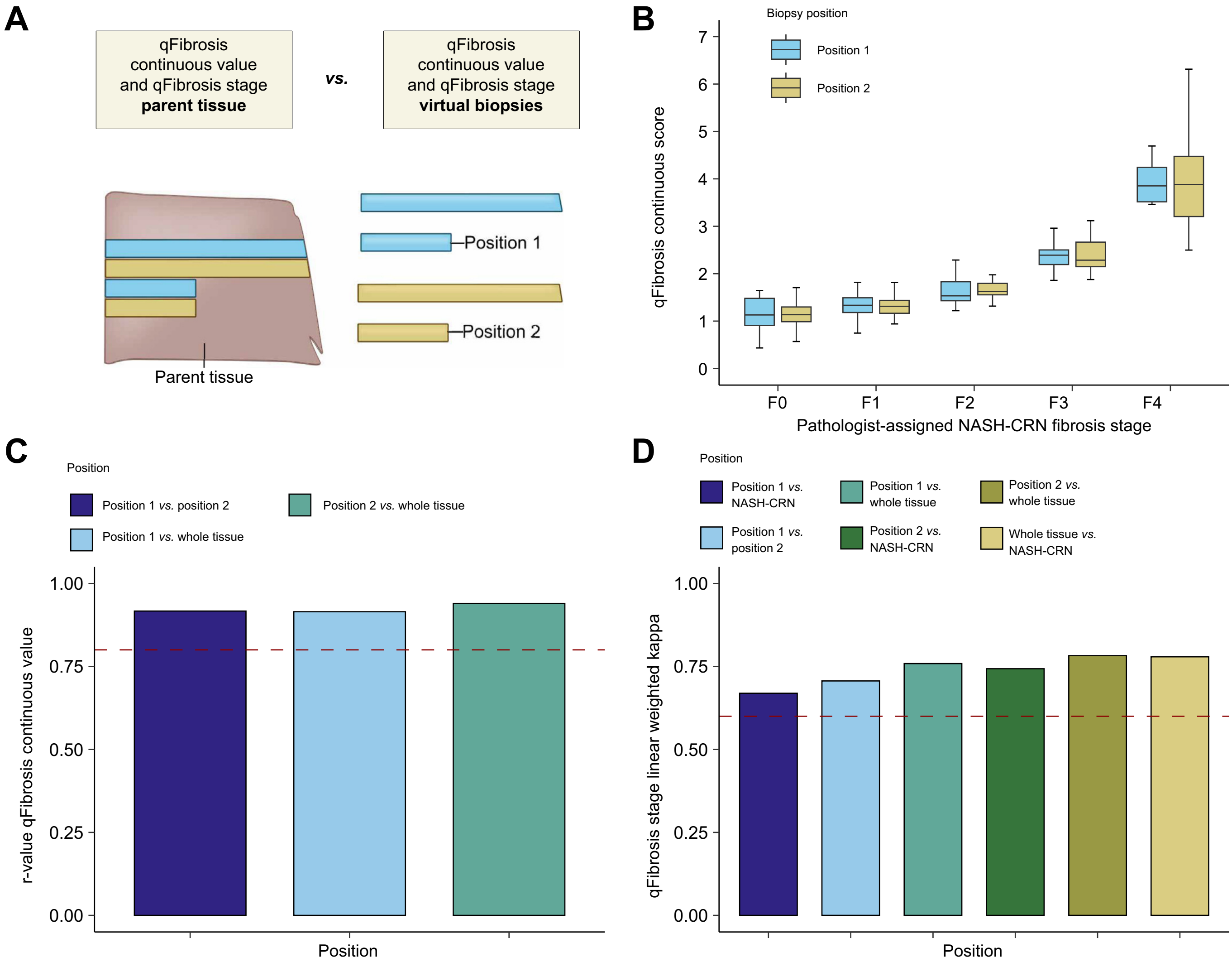

Fig. 5. Influence of adjacent position sampling variation on qFibrosis metrics. (A) Schematic of methodology for adjacent position variation. (B) qFibrosis continuous value between each pathologist-assigned NASH-CRN stage for adjacent positions 1 and 2. Tukey's fences method was used to exclude outliers from the plot. Boxplots are median with lower and upper hinges corresponding to the first and third quartiles, respectively, and whiskers extend to values no further than 1.5× IQR from the hinges. (C) Spearman's rank correlation coefficient of the comparison between qFibrosis continuous value derived from adjacent positional variations 1 and 2 and that derived from the whole parent section. Red dotted line at Spearman's correlation coefficient r_s = 0.8. (D) Linear weighted kappa values comparing qFibrosis stage derived from adjacent virtual core biopsy positions 1 and 2 and the NASH-CRN stage from the whole parent section. Red dotted line at a linear weighted kappa = 0.6. NASH-CRN, Non-alcoholic Steatohepatitis Clinical Research Network.

When the sampled area for virtual core biopsies was rotated relative to the first sample position (Fig. 6A and B), Spearman's coefficient for the correlation between qFibrosis continuous values derived from the whole parent section and virtual core biopsies were all between 0.90 and 0.95, demonstrating that the qFibrosis continuous values were equally consistent across a range of rotations (Fig. 6C). The linear weighted kappa values for the agreement between the qFibrosis stage derived from the virtual core biopsies and whole parent section were between 0.70 and 0.81 without an evident pattern of variation (Fig. 6D).

Overall, these results suggest that assessment by SHG/TPEF imaging with qFibrosis is robust to the origin of assessed tissue within the organ, ensuring the reliability and reproducibility of fibrosis quantification across different biopsy configurations.

Discussion

The present study evaluated the impact of liver biopsy length, width, and position on fibrosis quantification in MASLD using SHG/TPEF with AI-assisted analysis. Such analysis has recently been shown to help pathologists reach greater consensus when scoring fibrosis stage, and our findings inform on optimal biopsy standards necessary for clinical trials using such techniques.[14] We used the virtual core sampling methodology developed by Bedossa *et al.*[22] to evaluate the impact of sampling variability on fibrosis assessment in chronic hepatitis C and applied it to biopsies from MASLD. We show that extending the length and width of virtual core biopsies improves their concordance with the 'ground truth' computational fibrosis metrics and pathologist-assigned NASH-CRN stage,

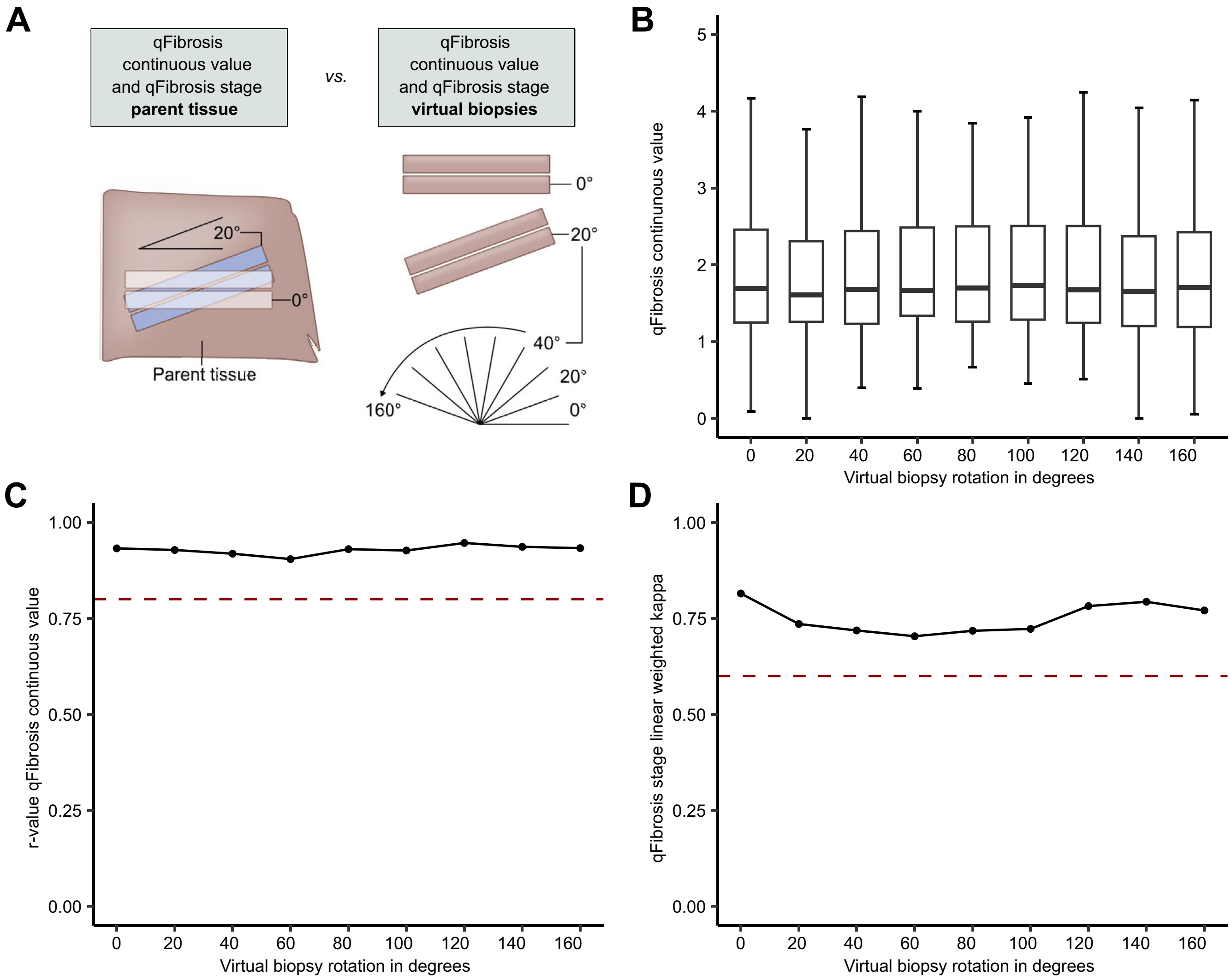

Fig. 6. Influence of rotational position sampling variation on qFibrosis metrics. (A) Schematic of the methodology for rotation position variation. (B) qFibrosis continuous value across each of the rotations. Tukey's fences method was used to exclude outliers from the plot. Boxplots are median with lower and upper hinges corresponding to the first and third quartiles, respectively, and whiskers extend to values no further than 1.5× IQR from the hinges. (C) Spearman's rank correlation coefficient of qFibrosis continuous value across the rotational variations compared with the value derived from the whole parent section. Red dotted line at r_s = 0.8. (D) Linear weighted kappa scores for qFibrosis stage across the rotational variations in virtual core biopsy origin compared with the NASH-CRN stage from the whole parent section. Red dotted line at the linear weighted kappa = 0.6. NASH-CRN, Non-alcoholic Steatohepatitis Clinical Research Network.

up to an optimal length of approximately 20–26 mm and width of 0.9–1.0 mm, depending on the evaluation metric. Importantly, biopsies that exceed these dimensions provide only limited additional accuracy.

Traditional subjective ordinal staging of fibrosis is a central requirement in clinical trials evaluating treatments for MASH. However, it has been well documented that such subjective scoring is compromised by inherent intra- and inter-observer variability. The high screen failure rates in clinical trials for MASH, where patients are required to have F2/F3 fibrosis stage for inclusion, or inclusion of patients who may not have met inclusion criteria on subsequent review have been suggested as a consequence of such variation[24] and that this may account, in part, for the failure of so many trials in MASH.[25] In semaglutide trials, the endpoint of improvement in fibrosis score in the phase II trial[26] was not met, but the endpoint was met in the larger phase III ESSENCE trial, suggesting that ordinal scoring, however expertly undertaken, was ill-suited to identifying changes in small cohorts. Further, the wisdom of coercing the dynamic, bidirectional process of scar formation and resolution into a limited number of ordinal categories has always been questionable. The assessment of scarring in whole-slide images of stained or unstained liver core biopsy sections using computational means removes observer-related variation and generates continuous metrics, offering a potential solution to the two most significant flaws in traditional subjective fibrosis staging. Recent data have shown that SHG/TPEF tools help expert pathologists reach significantly greater agreement when scoring fibrosis.[14] Generation of a continuous value also allows subtle changes in fibrosis that pathologists can readily observe but are not captured by the existing ordinal scoring systems, or potentially even changes that may be inapparent on conventional collagen staining, to be identified and compared in a way that offers greater insights into the

fibrospatial signatures of disease progression or treatment response. For example, analysis of liver biopsies from patients in the FLIGHT-FXR study showed that a reduction or an increase in qFibrosis parameters was demonstrable in cases where there is no change in the ordinal pathologist-assigned fibrosis score after treatment with tropifexor.[27]

However, although practice guidelines have defined liver core biopsy adequacy based on size for many years, with some data including that generated by the same methodology[22] to support these minimum standards, there are little data concerning the adequacy of biopsies specific to stain-free computational assessment of liver fibrosis generally or in MASH specifically to support the definition of any minimum core biopsy standard. It is illogical to use computational tools to overcome shortcomings in subjective assessment but not to define the circumstances when these should be applied cogently. Our data provide a basis for the establishment of such standards when quantifying liver fibrosis in MASH using SHG/TPEF and are in keeping with the current literature assessing the impact of core biopsy length on traditional subjective assessment, as well as existing clinical practice guidelines for core biopsy adequacy.[15–17,22] International guidelines from the Royal Colleges of Radiologists and Pathologists, the British Society of Gastroenterology, EASL-ALEH, and the AASLD recommend a biopsy length of between 20 and 25 mm using a 16G needle.[28–30]

A needle core liver biopsy represents a small sample relative to a large organ, and a single 4-µm-thick section represents a small sample of this core. The limitations of such sampling are well recognised but accepted, given that only the proteins, cells, and tissue in hand allow for the refined assessment, interpretation, and characterisation made possible through traditional histological examination and/or computational assessment of tissue sections. A concern, which could be seen as an extreme interpretation, is that requiring an additional, likely sequential, unstained section from the same block for SHG/TPEF imaging might introduce variations compared with the adjacent matrix-stained section. This view assumes that the matrix-stained section is the definitive reference, and that data from the unstained section should correspond exactly to it. However, we have shown that adjacent virtual cores, analogous to sequential sections in a different tissue plane, when assessed by SHG/TPEF imaging, yield equivalent information. In addition, the qFibrosis metrics showed high degrees of agreement and correlation between the whole parent section and the virtual biopsies, regardless of biopsy orientation. One study has previously investigated the effects of biopsy needle direction in transjugular liver biopsies and found that certain needle orientations produced longer cores with more complete portal tracts.[31] Similar studies have not been performed in percutaneous liver biopsies, and, regardless, the qFibrosis metrics show robust performance despite variation in the orientation.

Other authors have previously made recommendations that sample adequacy be reported in clinical trials.[24,32,33] According to one systematic review, a large proportion of clinical trials in hepatology do not report on the biopsy length, and of those that do, a large proportion fail to obtain a mean length at or above the recommended value.[18] At present, unless agreed by the sponsor, such metrics are not routinely provided. A recent pivotal phase III trial of resmetirom included a protocol requirement for obtaining a core of 20 mm or greater using a 16G cutting needle.[10] However, the mechanism of auditing this requirement was not described, and sampling variation data was not provided. The Royal College of Pathologists' clinical guidelines recommend a three-tier quality assurance system: good (total core length >20 mm), compromised (10–20 mm), and inadequate (<10 mm).[28] We also advocate for the routine reporting of biopsy length and width in prospective studies using the qFibrosis continuous value and stage or other computational pathology tools. This information is a key element of any clinical pathology report and can be easily calculated computationally, where such digital pathology methods are used.

Our study has inherent limitations. The virtual biopsy was automatically generated using rectangular cropping of the tissue, which may not accurately replicate the natural variability of the biopsy edges seen in true native biopsies, although this is the robust methodology previously used in the context of Picrosirius Red-stained sections and chronic HCV infection to generate data that have been used in many practice guidelines. The impact of the liver resection block location (*i.e.* subcapsular *vs*. deep or left *vs*. right lobe) on variability was also not analysed, restricting our ability to fully address location-related heterogeneity of MASH-associated pathology. The study was explicitly restricted to MASLD, and so data informing the use of the same adequacy criteria in the assessment of scarring using SHG/TPEF in other aetiologies are lacking.

Affiliations

[1]*Edinburgh Pathology, University of Edinburgh, Edinburgh, UK;* [2]*Histoindex Pte. Ltd., Singapore;* [3]*Division of Medicine, University College London, London, UK;* [4]*Laboratory of Pathology, Center for Cancer Research, National Cancer Institute, National Institutes of Health, Bethesda, MA, USA;* [5]*Centre for Inflammation Research, Institute for Regeneration and Repair, University of Edinburgh, Edinburgh, UK;* [6]*Stravitz-Sanyal Institute of Liver Disease and Metabolic Health, Virginia Commonwealth University School of Medicine, Richmond, VA, USA*

Abbreviations

AI, artificial intelligence; CoV, coefficient of variation; EASL-ALEH, European Association for the Study of the Liver–Asociacion Latinoamericana para el Estudio del Higado; MASH, metabolic dysfunction-associated steatohepatitis; MASLD, metabolic dysfunction-associated steatotic liver disease; NASH-CRN, non-alcoholic Steatohepatitis Clinical Research Network; PSR, Picrosirius Red; SHG, second-harmonic generation; TPEF, two-photon excitation fluorescence.

Financial support

This research was funded in part by Innovate UK Eureka (Reference: 105976). Establishment of the SteatoSITE multimodal MASLD resource was funded by Innovate UK (Reference: TS/R017581/1). For the purpose of open access, the authors have applied a creative commons attribution (CC BY) licence to any author-accepted manuscript version arising.

Conflicts of interest

YR, KA, EC, and DT are employees of HistoIndex. DT is a stockholder in HistoIndex. JAF serves as a consultant or an advisory board member for Stellaris, Resolution Therapeutics, Kynos Therapeutics, Ipsen, River 2 Renal Corp., Stimuliver, Global Clinical Trial Partners, and Guidepoint and has received speakers' fees from Resolution Therapeutics and HistoIndex and research grant funding from GlaxoSmithKline and Genentech. TJK serves as a consultant or an advisory board member for Resolution Therapeutics, Clinnovate Health, HistoIndex, Fibrofind, Kynos Therapeutics, Perspectum, Concept Life Sciences, and Jazz

Pharmaceuticals and has received speakers' fees from Servier Laboratories, Jazz Pharmaceuticals, Astrazeneca, HistoIndex, and Incyte Corporation. DTF has no conflicts of interest to declare. NVN is an advisor to HistoIndex. DEK has uncompensated collaborations with HistoIndex and HighTide. AJS has stock options in Genfit, Akarna, Tiziana, Rivus, Indalo, Durect Inversago, and Galmed. He has served as a consultant for Astrazeneca, Salix, Tobira, Takeda, Akero, 89 Bio, Boston Pharma, Intercept, Genentech, PathAI, HistoIndex, Alnylam, Regeneron, Hanmi, LG Chem, Aligos, Altimmune, Gilead, Terns, Merck, Boehringer Ingelheim, Eli Lilly, Novartis, Novo Nordisk, Pfizer, Surrozen, Poxel, and Zydus. His institution has received grant support from Gilead, Avant Sante, Salix, Tobira, Bristol Myers, Shire, Intercept, Merck, Astra Zeneca, Malinckrodt, Cumberland, and Novartis. Furthermore, he receives royalties from Elsevier and UptoDate.

Please refer to the accompanying ICMJE disclosure forms for further details.

Authors' contributions

Conceptualised and planned the research: AJS, DEK, NVN, EC, DT. Contributed to the study design: all authors. Provided annotated patient samples: TJK, JAF. Applied the computational algorithms, acquired the data, and performed statistical analysis: YR, KA. Contributed to analysis and validation of the data and interpretation of the research: all authors. Wrote the original draft manuscript and created data visualisations: DTF. Revised the manuscript: TJK, JAF. Reviewed original and subsequent drafts, provided feedback, and approved the final version of the manuscript: all authors.

Data availability statement

All data used for analyses in this study are available from the corresponding author upon reasonable request.

Acknowledgements

We are grateful to L. McMahon and M. McNeil (Precision Medicine Scotland—Innovation Centre) for project management, data compliance and security, and workspace provision. We thank J.J. Li for her assistance in data acquisition and cleaning.

Supplementary data

Supplementary data to this article can be found online at https://doi.org/10.1016/j.jhepr.2025.101449.

References

Author names in bold designate shared co-first authorship

[1] Younossi ZM, Golabi P, Paik JM, et al. The global epidemiology of nonalcoholic fatty liver disease (NAFLD) and nonalcoholic steatohepatitis (NASH): a systematic review. Hepatology 2023;77:1335–1347.

[2] Lekakis V, Papatheodoridis GV. Natural history of metabolic dysfunction-associated steatotic liver disease. Eur J Intern Med 2024;122:3–10.

[3] McHutchison J, Poynard T, Afdhal N. Fibrosis as an end point for clinical trials in liver disease: a report of the International Fibrosis Group. Clin Gastroenterol Hepatol 2006;4:1214–1220.e1.

[4] Cheung A, Neuschwander-Tetri BA, Kleiner DE, et al. Defining improvement in nonalcoholic steatohepatitis for treatment trial endpoints: recommendations from the Liver Forum. Hepatology 2019;70:1841–1855.

[5] Pavlides M, Birks J, Fryer E, et al. Interobserver variability in histologic evaluation of liver fibrosis using categorical and quantitative scores. Am J Clin Pathol 2017;147:364–369.

[6] Serdjebi C, Bertotti K, Huang P, et al. Automated whole slide image analysis for a translational quantification of liver fibrosis. Sci Rep 2022;12:17935.

[7] Gawrieh S, Sethunath D, Cummings OW, et al. Automated quantification and architectural pattern detection of hepatic fibrosis in NAFLD. Ann Diagn Pathol 2020;47:151518.

[8] Sun W, Chang S, Tai DCS, et al. Nonlinear optical microscopy: use of second harmonic generation and two-photon microscopy for automated quantitative liver fibrosis studies. J Biomed Opt 2008;13:064010.

[9] **Xu S**, **Wang Y**, Tai DCS, et al. *q*Fibrosis: a fully-quantitative innovative method incorporating histological features to facilitate accurate fibrosis scoring in animal model and chronic hepatitis B patients. J Hepatol 2014;61:260–269.

[10] Harrison SA, Bedossa P, Guy CD, et al. A phase 3, randomized, controlled trial of resmetirom in NASH with liver fibrosis. N Engl J Med 2024;390:497–509.

[11] Sanyal AJ, Lopez P, Lawitz EJ, et al. Tropifexor for nonalcoholic steatohepatitis: an adaptive, randomized, placebo-controlled phase 2a/b trial. Nat Med 2023;29:392–400.

[12] Sun Y, Zhou J, Wu X, et al. Quantitative assessment of liver fibrosis (qFibrosis) reveals precise outcomes in Ishak "stable" patients on anti-HBV therapy. Sci Rep 2018;8:2989.

[13] Kendall TJ, Chng E, Ren Y, et al. Outcome prediction in metabolic dysfunction-associated steatotic liver disease using stain-free digital pathological assessment. Liver Int 2024;44:2511–2516.

[14] Abdurrachim D, Lek S, Lin Ong CZ, et al. Utility of AI digital pathology as an aid for pathologists scoring fibrosis in MASH. J Hepatol 2025;82:898–908.

[15] Vuppalanchi R, Ünalp A, Van Natta ML, et al. Effects of liver biopsy sample length and number of readings on sampling variability in nonalcoholic fatty liver disease. Clin Gastroenterol Hepatol 2009;7:481–486.

[16] Ratziu V, Charlotte F, Heurtier A, et al. Sampling variability of liver biopsy in nonalcoholic fatty liver disease. Gastroenterology 2005;128:1898–1906.

[17] Colloredo G, Guido M, Sonzogni A, et al. Impact of liver biopsy size on histological evaluation of chronic viral hepatitis: the smaller the sample, the milder the disease. J Hepatol 2003;39:239–244.

[18] Cholongitas E, Senzolo M, Standish R, et al. A systematic review of the quality of liver biopsy specimens. Am J Clin Pathol 2006;125:710–721.

[19] **Kendall TJ**, **Jimenez-Ramos M**, Turner F, et al. An integrated gene-to-outcome multimodal database for metabolic dysfunction-associated steatotic liver disease. Nat Med 2023;29:2939–2953.

[20] **Liu F**, **Goh GB-B**, Tiniakos D, et al. qFIBS: an automated technique for quantitative evaluation of fibrosis, inflammation, ballooning, and steatosis in patients with nonalcoholic steatohepatitis. Hepatology 2020;71:1953–1966.

[21] Chang PE, Goh GBB, Leow WQ, et al. Second harmonic generation microscopy provides accurate automated staging of liver fibrosis in patients with non-alcoholic fatty liver disease. PLoS One 2018;13:e0199166.

[22] Bedossa P, Dargère D, Paradis V. Sampling variability of liver fibrosis in chronic hepatitis C. Hepatology 2003;38:1449–1457.

[23] Palmer T, Georgiades I, Treanor D, et al. Improved tissue sections for medical liver biopsies: a comparison of 16 vs 18 g biopsy needles using digital pathology. J Clin Pathol 2014;67:415–419.

[24] Davison BA, Harrison SA, Cotter G, et al. Suboptimal reliability of liver biopsy evaluation has implications for randomized clinical trials. J Hepatol 2020;73:1322–1332.

[25] Harrison SA, Allen AM, Dubourg J, et al. Challenges and opportunities in NASH drug development. Nat Med 2023;29:562–573.

[26] Loomba R, Abdelmalek MF, Armstrong MJ, et al. Semaglutide 2·4 mg once weekly in patients with non-alcoholic steatohepatitis-related cirrhosis: a randomised, placebo-controlled phase 2 trial. Lancet Gastroenterol Hepatol 2023;8:511–522.

[27] Naoumov NV, Brees D, Loeffler J, et al. Digital pathology with artificial intelligence analyses provides greater insights into treatment-induced fibrosis regression in NASH. J Hepatol 2022;77:1399–1409.

[28] Neuberger J, Patel J, Caldwell H, et al. Guidelines on the use of liver biopsy in clinical practice from the British Society of Gastroenterology, the Royal College of Radiologists and the Royal College of Pathology. Gut 2020;69:1382–1403.

[29] European Association for Study of Liver, Asociacion Latinoamericana para el Estudio del Higado. EASL-ALEH Clinical Practice Guidelines: non-invasive tests for evaluation of liver disease severity and prognosis. J Hepatol 2015;63:237–264.

[30] Sanyal AJ, Friedman SL, McCullough AJ, et al. Challenges and opportunities in drug and biomarker development for nonalcoholic steatohepatitis: findings and recommendations from an American Association for the Study of Liver Diseases–U.S. Food and Drug Administration Joint Workshop. Hepatology 2015;61:1392.

[31] Clark TWI, McCann JW, Salsamendi J, et al. Optimizing needle direction during transjugular liver biopsy provides superior biopsy specimens. Cardiovasc Intervent Radiol 2014;37:1540–1545.

[32] Langford CR, Goldinger MH, Treanor D, et al. Improved pathology reporting in NAFLD/NASH for clinical trials. J Clin Pathol 2022;75: 73–75.

[33] Filozof CM, Lackner C, Romero-Gómez M, et al. Best practices in liver biopsy histologic assessment for nonalcoholic steatohepatitis clinical trials: expert opinion. GastroHep 2022;2022:3538103.

Keywords: Humans; Metabolic dysfunction-associated steatotic liver disease; Non-alcoholic fatty liver disease; Liver cirrhosis; Biopsy; Artificial intelligence; Deep learning; Second-harmonic generation microscopy; Quality control; Collagen.

Received 17 March 2025; received in revised form 24 April 2025; accepted 29 April 2025; Available online 8 May 2025

Journal of Hepatology, Volume *7*

Supplemental information

Effect of liver biopsy size on MASLD fibrosis assessment by second-harmonic generation/two-photon excitation fluorescence microscopy

Daniel T. Field, Yayun Ren, Kutbuddin Akbary, Elaine Chng, Dean Tai, Nikolai V. Naoumov, David E. Kleiner, Jonathan A. Fallowfield, Timothy J. Kendall, and Arun J. Sanyal

Effect of liver biopsy size on MASLD fibrosis assessment by second harmonic generation/two-photon excitation fluorescence microscopy

Daniel T. Field, Yayun Ren, Kutbuddin Akbary, Elaine Chng, Dean Tai, Nikolai V. Naoumov, David E. Kleiner, Jonathan A. Fallowfield, Timothy J. Kendall, Arun J. Sanyal

Table of contents

Fig. S1……………………………………………………………………………2

Fig. S2……………………………………………………………………………3

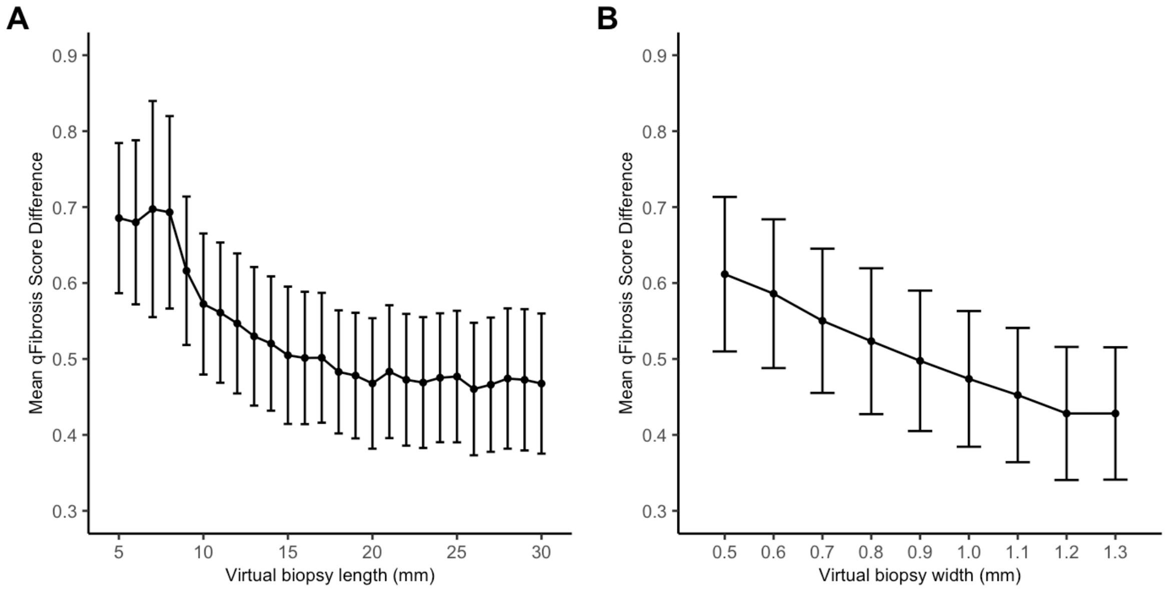

***Fig. S1. qFibrosis continuous value mean absolute differences between virtual biopsies and whole parent section**. (A) Across length variation. (B) Across width variation. Mean with standard error of the mean.*

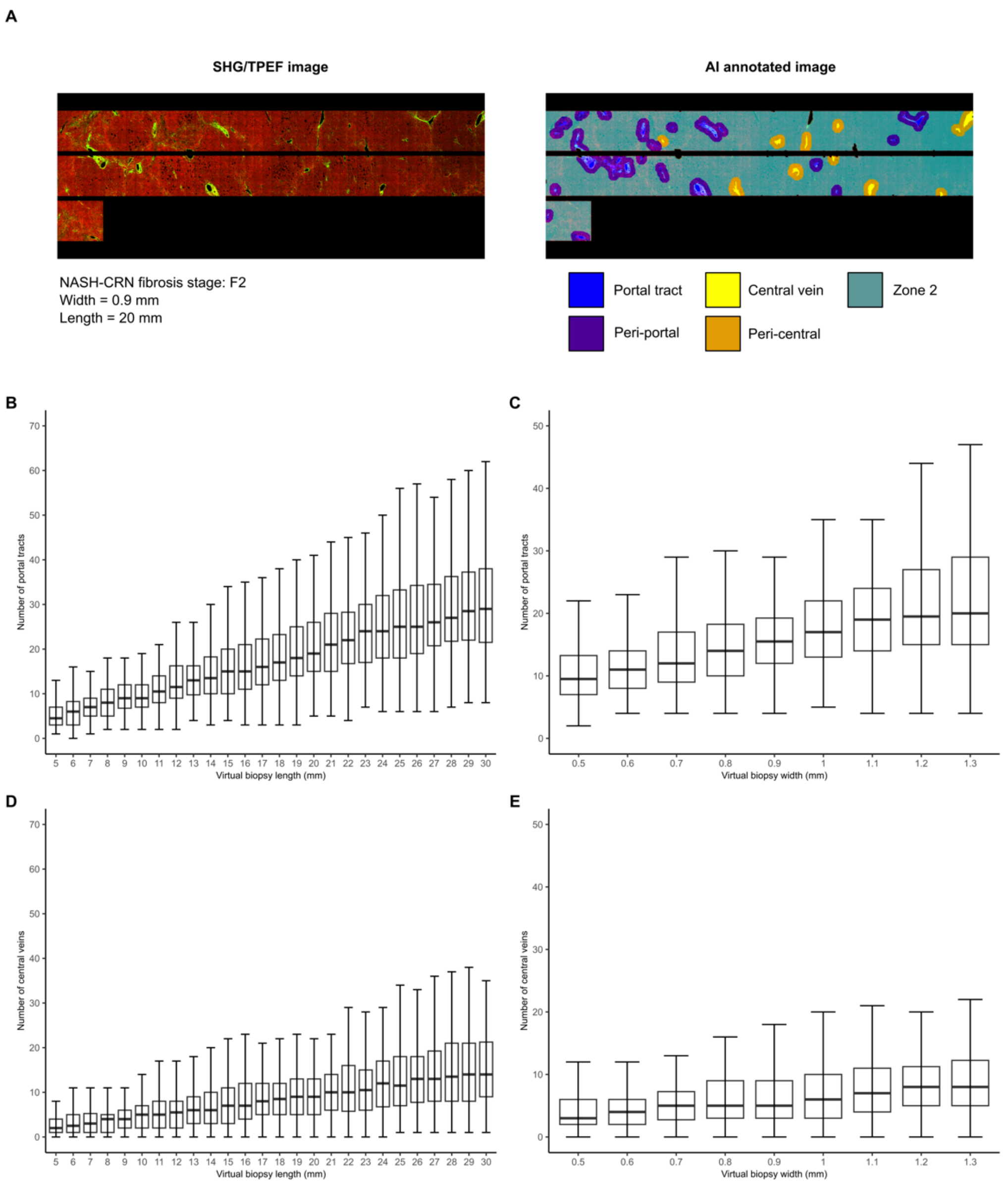

Fig. S2. Portal tract and central vein number in virtual biopsies from cases across all pathologist-assigned NASH-CRN stages. *(A) AI segmentation of central vein and portal vein areas. (B) Portal tracts across length variation. (C) Portal tracts across width variation. (D) Central veins across length variation. (E) central veins across width variation. All boxplots are median with lower and upper hinges corresponding to the first and third quartiles, whiskers extend to values no further than 1.5 * inter-quartile range from the hinge.*
